# Implications of CD154 and Its Receptors in the Pathogenesis and Treatment of Systemic Lupus Erythematosus

**DOI:** 10.3390/cells13191621

**Published:** 2024-09-28

**Authors:** Catherine Cornet Allard, Suzanne Salti, Walid Mourad, Ghada S. Hassan

**Affiliations:** Laboratoire d’Immunologie Cellulaire et Moléculaire, Centre de Recherche du Centre Hospitalier de l’Université de Montréal (CR-CHUM), 900 Rue Saint-Denis, Tour Viger, Montréal, QC H2X 0A9, Canada; catherine.cornet.allard@umontreal.ca (C.C.A.); suzanne.salti@umontreal.ca (S.S.)

**Keywords:** CD154, systemic lupus erythematosus, CD40, integrins, inflammation, apoptosis, antagonistic antibodies, thromboembolic complications

## Abstract

CD154, also known as CD40 ligand, is a costimulatory molecule involved in humoral and adaptive immune responses upon pairing with its classical receptor, CD40. The CD154/CD40 dyad is a key participant in the pathogenesis of many autoimmune diseases, including systemic lupus erythematosus (SLE). In SLE, the major cells at play, T and B lymphocytes, are shown to overexpress CD154 and CD40, respectively. Subsequently, these cells and other CD40-positive cells engage in numerous effector functions contributing to SLE development. With the recent identification of additional receptors for CD154, all belonging to the integrin family, the role of CD154 in SLE is more complex and calls for deeper investigation into its biological significance. Many therapeutic strategies directed against the CD154/CD40 couple have been deployed for the treatment of SLE and proved efficient in animal models and human studies. However, the incidence of thromboembolic complications in patients treated with these anti-CD154/CD40 antibodies halted their further clinical assessments and called for another class of therapies targeting these molecules. Second-generation antibodies directed against CD154 or CD40 are showing promising results in the advanced stages of clinical testing. Our review presents a thorough description of CD154 and its receptors, CD40 and the integrin family members in SLE pathogenesis. All these elements of the CD154 system represent important therapeutic targets for the treatment of SLE.

## 1. Introduction

Systemic lupus erythematosus (SLE) is a chronic multisystem autoimmune disease that mostly affects women of reproductive age, especially of African American, Hispanic, or Asian ethnicity [1]. As an autoimmune condition, SLE pathogenesis is characterized by the abnormal and excessive activity of the immune system, especially B and T lymphocytes, against normal cells and tissues of the body. Indeed, many auto-antibodies, especially antinuclear ones, can be found in the sera of SLE patients. The loss of tolerance against self-antigens and their recognition by antibodies (Abs) leads to the production of immune complexes, complement activation, cytokine production, and inflammation, which together result in different clinical presentations of SLE and organ manifestations, the most common being lupus nephritis (LN) [2].

The co-stimulatory molecule, CD154, and its receptor CD40, have gained increasing interest as important players in the different phases of SLE and have revealed themselves as promising therapeutic targets for disease treatment [3]. This review will provide a thorough description of the physiological effects of CD154 through its interactions with its long-time known receptor, CD40, and the biological significance of the CD154/CD40 couple in SLE initiation and development. We will also describe the interaction of CD154 with its more recently identified receptors, which all belong to the integrin family [4,5,6,7,8]. Such a global consideration of CD154 may allow for a more thorough understanding of its implication at different levels of SLE pathogenesis. In addition, an overview of previous and more recent therapeutic strategies involving the CD154 system in the treatment of SLE is presented herein.

## 2. CD154

Human CD154, CD40 L or gp39, is a 33–39 kDa type II transmembrane glycoprotein of the tumor necrosis factor (TNF) family. It is expressed in a transient fashion on the surface of activated T cells and platelets as well as basophils and eosinophils [9]. The CD154 molecule is an important effector in innate and adaptive immunity [9].

Along with its membrane-bound form (mCD154), CD154 also exists as a soluble molecule (sCD154), composed of residues 113 to 261 released from activated T cells or platelets [10,11]. Indeed, membrane-bound CD154 was shown to be proteolytically cleaved from the surface of activated T cells and/or platelets. Studies have revealed the role of MMP-2 and/or MMP-9 in catalyzing the cleavage of CD154 from the surface of activated platelets upon its binding to CD40 or to αIIbβ3 [12,13,14,15]. On the other hand, in activated T cells, such cleavage involves the metalloproteinases ADAM10 and ADAM17 following the binding of CD154 to CD40 [16]. Interestingly, mCD154 can undergo a spontaneous type of release from intracellular milieu or from the surface of activated T cells, also in an ADAM10/17-dependent manner [10,11]. Although the biological effects of sCD154 are still incompletely understood, it is thought that the liberation of sCD154, following the interaction of mCD154 with CD40, allows for a reduction in the resulting response. In fact, studies have shown that in comparison to sCD154, mCD154 produces a more potent effect upon binding to CD40-expressing cells [11,17,18]. In addition, data from our laboratory have outlined the importance of the soluble form of CD154 for a proper interaction with its integrin receptors, as reported with the α5β1 and αMβ2 integrins [11,19].

Similarly to other members of the TNF family, membrane-bound or soluble CD154 exist as non-covalently bound homotrimers, a pre-requisite of biological activity [20,21]. This homotrimeric structure may also allow CD154 to interact with more than one receptor at once if concomitantly expressed on the same cell [6]. This possibility could create a wide diversity of CD154-mediated responses.

## 3. CD154 Receptors

Although for almost two decades CD40 was thought to be the only CD154 receptor, several studies have demonstrated that sCD154 may bind to several members of the integrin family such as αIIbβ3, αMβ2, α5β1, αvβ3 and α4β1 integrins [4,5,6,7,8].

### 3.1. The CD40 Molecule

CD40, the classical receptor of CD154, is a 45–50 kDa phosphorylated type I membrane glycoprotein belonging to the family of tumor necrosis factor receptors (TNFRs) [9,21]. It is constitutively expressed on many cells, including antigen presenting cells (APCs) such as B lymphocytes, dendritic cells (DCs), monocytes, macrophages as well as platelets, fibroblasts, epithelial and endothelial cells, and smooth muscle cells [9,21,22]. Our group demonstrated an interesting feature of CD40, whereby it undertakes homodimerization at its cysteine residues in position 238, a process of high significance in some CD40-mediated biological functions [23]. Because its cytoplasmic region has no enzymatic activity, CD40 associates with adaptor molecules, known as TRAFs (TNF receptor-associated factors) [22]. Upon CD154-CD40 interaction and the recruitment of TRAFs, including TRAF 1, 2, 3, 5 and 6, many signaling pathways are initiated. These include the activation of nuclear factor κ B (NFκB), phosphatidylionositol-3 kinase (PI-3K), c-Jun-n-terminal-kinase (JNK), and mitogen-activated protein kinases (MAPK) p38 and extracellular signal-regulated kinases 1/2 (ERK1/2), etc. [24].

The CD154-CD40 couple is implicated in the regulation of humoral as well as cell-mediated immune responses. In this context, it has been shown that the CD154/CD40 interaction results in bidirectional signaling simultaneously activating CD154- and CD40-expressing cells [11,23]. In B cells, CD40 signaling allows the expression of costimulatory molecules such as B7-1 (CD80) and -2 (CD86), promoting what is known as T cell-dependent B cell responses [25,26]. CD40 ligation plays a crucial role in the proliferation of B cells, the formation of germinal centers, isotype switching, production of memory B cells, as well as the liberation of cytokines and cytotoxic radicals by these lymphocytes [27,28]. Additionally, the CD154-CD40 couple acts on macrophages, monocytes, DCs, fibroblasts and endothelial cells (ECs), inducing their proliferation, expression of costimulatory and adhesion molecules, as well as secretion of pro-inflammatory cytokines [28,29]. As for T cells, studies in murine models demonstrated that signaling via CD154 induces T cell priming as well as the proliferation of CD4^+^ and CD8^+^ T cells [30,31]. Also, as shown by our group, the ligation of CD154 on the surface of activated T cells triggers their release of various cytokines, including interleukin- (IL)-2 [23]. This plethora of effects in humoral and cell-mediated immunity underscores the implication of the CD154-CD40 costimulatory pair in the pathogenesis of multiple chronic inflammatory and autoimmune diseases. However, while CD154 was initially identified as the ligand of CD40 through mediating effects of the latter in various immune and non-immune cells, recent studies have identified additional functions of CD154 as a ligand of other surface molecules. Indeed, and as mentioned above, more receptors have been identified for CD154, all belonging to the integrin family [19], starting with the integrin αIIbβ3 [4], then integrins αMβ2 [5] and α5β1 [6], and lastly, integrins αvβ3 and α4β1 [7,8].

### 3.2. CD154 Receptors Belonging to the Integrin Family

The αIIbβ3 integrin, also termed GPIIb/IIIa is expressed on the surface of platelets and megakaryocytes and is known for its role in platelet aggregation via binding to ligands such as fibrinogen, fibronectin, and Von Willebrand factor [32]. In 2002, the CD154/αIIbβ3 interaction was first described and revealed to be important for the stabilization of the arterial thrombi [4,33] and also for inducing platelet activation and aggregation [34]. Interestingly, activating platelets via the CD154-αIIbβ3 binding enhanced an upregulation of their CD154 surface expression, a process of high significance in the development of atherosclerotic events by enhancing interactions between activated platelets and CD40-expressing ECs [35].

Another integrin, the αMβ2, also known as Mac-1, was identified as an additional receptor for CD154 [5]. This integrin is mainly found on the surface of monocytes, macrophages, granulocytes, and NK cells [36]. It binds to ligands such as vitronectin, fibrinogen, the complement fragment C3bi, intracellular adhesion molecule-1 (ICAM-1), and heparin and is involved in the pathogenesis of atherosclerosis by allowing the adhesion and rolling of myeloid cells on ECs and transendothelial migration [37]. Similarly, the interaction of αMβ2 with CD154 is also shown to induce monocyte adhesion and migration on ECs, thus enhancing the inflammatory process [5].

Yet additional members of the integrin family are being denoted as receptors for CD154, namely the α5β1 integrin [6]. Like αIIbβ3, α5β1 belongs to the RGD-binding subfamily of integrins. Its ligands include fibrinogen and fibronectin [32]. The α5β1 integrin is usually expressed on the surface of all nucleated cells [32,38]. Our observations revealed that sCD154 is capable of binding to CD40^−^/αIIbβ3^−^/α5β1^+^ monocytic cells in an α5β1-specific manner [6]. The biological significance of the CD154/α5β1 interaction will be further outlined below while describing the role of CD154 in inflammatory responses relating to SLE pathogenesis.

Adding to the list of integrins identified as receptors for CD154, Takada et al. revealed αvβ3, and later α4β1, as capable of binding CD154 [7,8]. Although little is known about the biological significance of these interactions, some studies suggested that CD154/αvβ3 might be implicated in tumorigenesis, inflammation, and atherosclerosis, while CD154/α4β1 could promote immune cell activation [7,8].

## 4. Role of CD154 in Systemic Lupus Erythematosus

As previously mentioned, many studies have demonstrated that CD154 plays an important role in many autoimmune diseases. From the activation of immune and non-immune cells to the induction of cell-mediated immunity and inflammation, CD154 is shown to highly contribute to the development and progression of autoimmunity. Numerous chronic inflammatory and autoimmune conditions are characterized by an enhanced expression of CD154 on T cells and of its classical receptor CD40 on other immune cells and various mesynchemal, endothelial, and epithelial cells. Inhibiting the interaction of the ligand with its receptor in animal models or human studies provided support to the pathogenic signature of the CD154/CD40 axis in numerous diseases of inflammatory and/or autoimmune nature, including rheumatoid arthritis (RA) [39], multiple sclerosis [40], autoimmune thyroiditis [41], polymyositis, dermatomyositis [42], inflammatory bowel diseases [43], and SLE. Our review will focus on describing the role of CD154, acting via its classical receptor, CD40, or its newly described receptors, members of the integrin family in SLE. The following sections provide an overview of the implications of CD154 at various stages of the disease.

### 4.1. The CD154-CD40 Dyad in SLE

CD154 is overexpressed on the CD4^+^ and CD8^+^ T lymphocytes of SLE patients. Indeed, studies have shown that activated CD4^+^ and CD8^+^ T cells of patients with active lupus or who are in remission for this condition express a higher level of CD154 than T cells of control individuals [44,45]. Additionally, studies also showed that the B cells of SLE patients and of BXBS mice affected by a lupus-like condition spontaneously express high levels of CD154 [44,46]. This abnormal CD154 expression has been linked to autoimmunity. Indeed, as demonstrated by Higuchi et al., the ectopic expression of CD154 on B cells of transgenic mice leads to the production of auto-antibodies and SLE symptoms such as glomerulonephritis [47]. Furthermore, in the spontaneous lupus model, BXBS mice, B cells ectopically expressing CD154 showed increased proliferation which could be halted by the administration of anti-CD40 Abs [46]. As in T and B lymphocytes, it is important to note the overexpression of CD154 on the monocytes of SLE patients, further highlighting the role of CD154-expressing myeloid cells in the pathogenesis of SLE [48].

Regarding the soluble counterpart of CD154, studies have demonstrated high concentrations of sCD154 in the sera of SLE patients, as compared to normal subjects with levels correlating to disease activity [49,50]. Soluble CD154 was shown to contribute to the expression of several immune accessory molecules, including CD54, CD95, and CD80 on B cells underscoring their activated state under SLE conditions [49]. Furthermore, the serum levels of sCD154 are increased in SLE patients having experienced thrombotic events and/or affected by secondary antiphospholipid syndrome [51]. Indeed, our team has previously revealed that sCD154 can induce platelet activation and aggregation through CD40-induced pathways [52,53].

**Figure 1:**
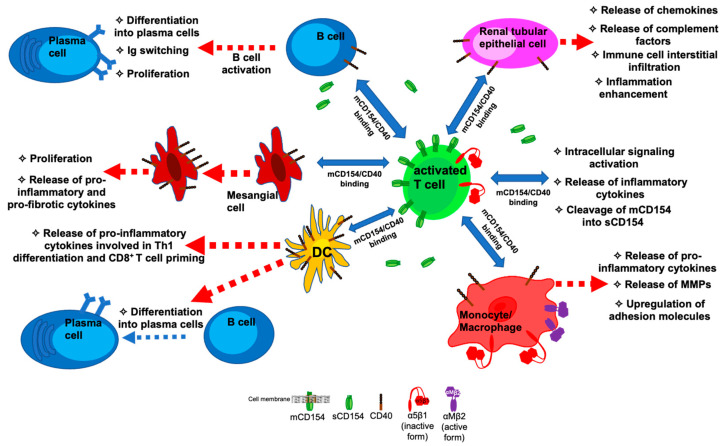
A model describing the bidirectional interaction of membrane-bound CD154 expressed on the surface of activated T cells with various CD40-positive cells in SLE pathogenesis.

In addition to the elevated levels of sCD154 in circulation and the overexpression of CD154 in multiple immune cells, CD40 is also upregulated on the surface of B cells and macrophages of SLE patients as well as on the endothelial and mesangial cells of the kidneys in those with class III and IV LN [3,54]. This increased presence of CD154 and CD40 on T cells, B cells, or other APCs of immune or non-immune nature is responsible for triggering cell activation and enhancing SLE progression and even potentiating complications and associated pathological conditions [55,56] (Figure 1). Indeed, the higher level of CD154 exhibited by SLE T cells [44,45] warrants the increased engagement of CD40 located on the surface of B cells, leading to the heightened expression of their costimulatory molecules, such as CD86, and their subsequent differentiation into auto-antibody-producing plasma cells [57,58]. In the same line of evidence, activating CD40 on the surface of germinal center B cells upregulates the expression of another costimulatory molecule, the inducible T cell costimulatory ligand (ICOS ligand), which, by interacting with its receptor (ICOS) on the surface of T follicular cells, further strengthens the T cell–B cell interaction, promoting Ab production. SLE B cells exhibited an overproduction of IgG upon co-culturing with activated autologous T cells, a response inhibited by anti-CD154 Abs [59]. Furthermore, at the level of the renal interstitium, auto-reactive B cells shown to overexpress CD40 (or even naïve ones) undergo proliferation and expansion and engage in auto-antibody production upon their interaction with CD154-expressing T cells [44]. In addition to B lymphocytes, other CD40-positive cells are targets of CD154 functions. CD154 originating from activated T cells or platelets was shown to promote the upregulation of CD40 on the surface of mesangial cells and thus enhance their proliferation and the release of pro-inflammatory factors, such as monocyte chemoattractant protein-1 (MCP-1), and pro-fibrotic factors, including TGF-β, which are important players in glomerular nephritis pathogenesis [60,61]. The interaction of CD154 on the surface of infiltrating T cells with CD40-positive renal tubular epithelial cells induces these latter to the secretion of various chemokines, such as regulated on activation, normal T-cell expressed and secreted (RANTES), MCP-1 and interferon (IFN)-γ-induced protein (IP)-10, as well as the C3 complement factor [56]. This enhances further immune cell interstitial infiltration and promotes inflammation and nephrogenesis. CD154 is also implicated in DC-mediated signaling and its role in various inflammatory events that underlie lupus development. A combined stimulus with Toll-like receptors (TLRs), IL-1 or IFN-γ, together with CD154, induces strong activation of DCs, enhancing the release of several pro-inflammatory cytokines such as IL-1 and IFN-γ themselves, IL-6, IL-12, IL-23, and IL-18, which promotes Th1 T cell differentiation as well as the priming of CD8^+^ T cells [62,63,64]. The role of CD40-activated DCs in lupus is further revealed via their contribution to B cell differentiation into Ab-producing plasma cells [65].

As mentioned above, CD154 on activated T cells interacting with CD40 on the surface of B cells or other APCs induces signaling pathways in a bidirectional manner, activating all cells in the equation, including T cells [28,29]. Indeed, our group and others have demonstrated that the co-stimulation of T cells via CD154 triggers intracellular signal activation and induces numerous T cell functions, including IL-2 production [23], IL-4 synthesis [66], and the cleavage of CD154 itself [11,16].

Furthermore, vascular events which are responsible for a high degree of morbidity in SLE patients, involve a significant contribution of the CD154-CD40 dyad acting at different phases in the vascular pathology [28,37,67]. Indeed, the CD154-CD40 couple was found to be highly expressed in atherosclerotic lesions. CD154-CD40 interactions between cells such as activated T lymphocytes and ECs, smooth muscle cells or macrophages lead to the upregulation of adhesion molecules and the release of cytokines, MMPs, and tissue factor, all of which contribute to atherosclerotic plaque formation. In this context, the administration of anti-CD154 Abs to mice lacking the low-density lipoprotein (LDL) receptor and fed a high-cholesterol diet reduced their atherosclerotic plaque size and instability [68]. CD40 constitutively expressed on platelets could also be a mean of their activation via its binding to CD154, inducing the release of their granules content as well as the activation of their αIIbβ3 integrin, further underscoring the role of the CD154/CD40 pair in vascular events [69], and thus SLE complications.

### 4.2. The CD154-Integrin Dyad in SLE

**Figure 2:**
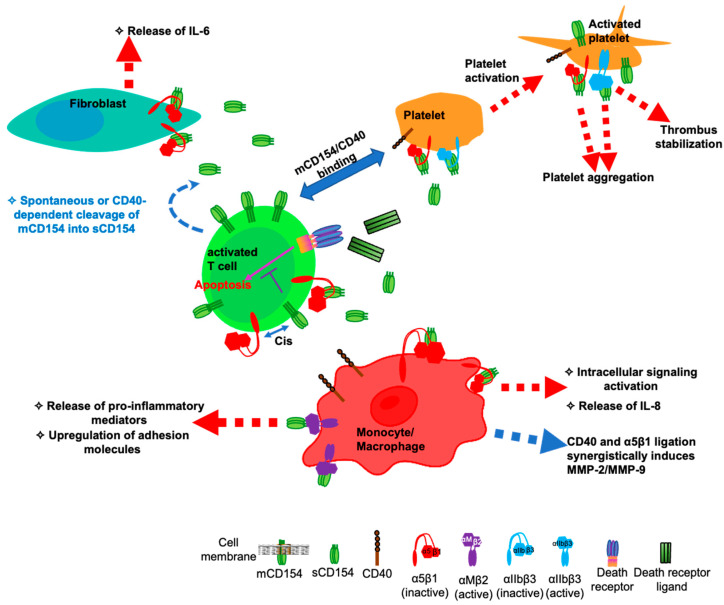
A model describing the interaction of soluble CD154 with members of the integrin family in SLE pathogenesis.

The discovery of novel receptors for CD154, all belonging to the integrin family opens new doors for a broader implication of CD154 in SLE pathogenesis. Although little information is available as to how the interaction of CD154 with these integrins may play a role in SLE, certain studies give us an insight into their potential influence in this context (Figure 2).

With respect to the α5β1 integrin as a receptor for CD154, new understandings have been elaborated demonstrating the possible implication of this dyad in the pathogenesis of autoimmune diseases such as SLE. Indeed, the CD154-α5β1 interaction plays an important role in inflammation. The ligation of CD154 to this integrin was shown to activate ERK1/2 signaling pathways in monocytes and their IL-8 production [6,70]. In addition, the binding of CD154 to α5β1 enhanced IL-6 release from the fibroblasts of asthmatic patients underscoring the role of such dyad in autoimmune responses such as allergy [71], and probably in other pathologies with an autoimmune characteristic such as SLE. Interestingly, a simultaneous ligation of α5β1 and CD40 was shown to result in a synergistic effect involving the activation of ERK1/2 and p38 signaling pathways as well as the production of MMP-2 and -9 [70], responses usually exhibited in arthritic inflammatory conditions such as SLE and RA [72,73].

Interestingly, a study by Nakayamada et al. showed that β1 integrin expression is increased on the T lymphocytes of patients with active SLE and that the activation of this receptor leads to the enhanced proliferation of T cells and the upregulation of their CD154 expression [74]. In the same line of evidence, our group has demonstrated that sCD154 interaction with α5β1 promotes T cell survival [75,76]. Upon binding to α5β1 on the surface of T cells, sCD154 was shown to inhibit T cell death induced by various death signals, including the Fas ligand, TRAIL, and TNF-α [75,76]. Altogether, these results suggest that the CD154/α5β1 dyad could contribute to the development and persistence of SLE by allowing the prolonged survival of effector T cells in this condition [19].

The biological significance of the CD154-α5β1 interaction was also revealed in promoting the activation and aggregation of platelets [4,77]. Therefore, and considering that both αIIbβ3/ and α5β1/CD154 dyads induce platelet activation and aggregation [77], it is possible that these pairs may be also contributing to thrombotic events associated with autoimmune and inflammatory pathologies such as SLE [4,28,77].

The role of the CD154/αMβ2 pair has been initially investigated in the context of vascular conditions and atherosclerosis by enhancing monocyte adhesion and migration, and the release of myeloperoxidase [5,37]. Nevertheless, such an inflammatory signature of the CD154/αMβ2 interaction could directly contribute to SLE pathogenesis.

Finally, the well-established notion of the trimeric structure of CD154 [20,78,79] and the more recent finding describing its interaction with its various receptors via distinct residues [80], highly suggest the capacity of CD154 to simultaneously bind more than one receptor and even potentially induce their cross-linking [78,79,80]. All these findings solicit a deeper investigation into the more complex role of CD154 in SLE.

## 5. Therapeutic Approaches in SLE

The current treatment arsenal for SLE includes a variety of immunomodulatory and immunosuppressive drugs [81]. Hydroxychloroquine, an antimalarial drug, is used in most cases of SLE and can be paired with other therapeutic agents such as NSAIDS, methotrexate, cyclophosphamide, azathioprine, and mycophenolate mofetil, in cases of mild to moderate disease severity [81]. In more severe cases, treatment often requires the use of systemic corticosteroids. Although these therapeutic strategies allow for a significant improvement in SLE prognosis, such use of immunosuppressive drugs can be associated with many adverse effects, which can take a toll on patients’ quality of life [82]. Many research efforts have been dedicated to the identification of specific biological agents for SLE treatment. Belimumab, a mAb directed against B cell-activating factor (BAFF), is the only biological currently approved for SLE treatment [83]. Rituximab, an anti-CD20 mAb, may also occasionally be used for patients with severe disease and who are not responding to other therapeutic avenues [84]. Considering that CD154 is an important effector in the pathogenesis of SLE, it has recently been subject to research concerning its potential use as a target for novel biological therapies in SLE.

### 5.1. Anti-CD154 and Anti-CD40 Agents in SLE Murine Models

The administration of anti-CD154 treatment in murine models of SLE was proven beneficial in many studies [85]. Indeed, Early et al. demonstrated that the administration of an anti-CD154 Ab to the spontaneous lupus-prone mice, New Zealand Black x New Zealand White (NZB/W), decreased their auto-antibody production and prolonged their survival. Responding mice showed no deposition of immune complexes in their renal glomeruli [86]. Another study evaluating early and late treatments with anti-CD154 Abs demonstrated similar efficiency in lupus-prone mice. Authors showed that treating pre-nephritic NZB/W F1 mice with anti-CD154 Abs resulted in a reduction in renal immune complex deposition, a response that persisted even after treatment halting. The administration of the same treatment to corresponding mice with established LN reduced their renal gene expression of pro-inflammatory and profibrotic factors, and induced remission in 40% of cases [87]. Similarly, Kalled et al. demonstrated that anti-CD154 Ab treatment of Swiss Webster x New Zealand Black (SWRxNZB) mice with established LN enhanced their survival and decreased their risk of severe nephritis [88]. Interestingly, treatment administered at younger age revealed better outcome than with older mice (7 months (mo) of age) which necessitated a more aggressive treatment strategy. Additionally, the concomitant administration of anti-CTLA4 and anti-CD154 mAbs was shown to delay SLE onset in NZB/W F1 lupus-prone mice and even to prolong the survival of previously treated mice with more advanced states of the disease [89].

As to anti-CD40 mAb therapies, it also showed numerous benefits in the treatment of lupus in mice models [3,90]. When comparing NZB/W F1 mice treated with a rat/mouse chimeric antagonistic anti-CD40 Ab, following the onset of renal damage to those treated with the broad-spectrum anti-inflammatory drug, prednisolone, data revealed both agents as capable of reducing the activation of immune cells in the germinal centers. However, only anti-CD40 promoted renal protection [91]. Anti-CD40 Ab significantly reversed the upregulation of inflammatory genes and the downregulation of metabolic pathways observed in kidneys of lupus mice to levels in control mice [91]. Interestingly, the same study demonstrated the efficiency of anti-CD40 in reducing inflammation in yet another model of spontaneous lupus, the MRL/lpr mouse. In spite of promising results obtained with the anti-CD40 Ab treatment, its effect was abrogated upon treatment cessation, unlike the long-lasting effect exhibited by the anti-CD154 Abs in lupus-prone mice, underlining the possible induction of tolerance in the latter case [3,91].

### 5.2. Anti-CD154 Agents in SLE Clinical Studies

Based on promising data obtained upon the CD154- or CD40- related treatment of lupus animals, numerous anti-CD154/CD40 agents compatible for use in humans were developed, as outlined in Table 1.

Two humanized anti-CD154 mAbs, Ruplizumab or BG9588 (Biogen Inc., Cambridge, MA, USA) and Toralizumab or IDEC-131 (Idec Pharmaceuticals, San Diego, CA, USA), were developed and tested in clinical trials.

Ruplizumab is a humanized anti-CD154 mAb composed of the complementary-determining regions of the 5c8 mAb (murine anti-human CD154), combined with human variable-region framework residues as well as IgG1 constant region [92]. A phase II clinical trial was conducted to evaluate the safety and efficacy of Ruplizumab in SLE. Indeed, patients with active LN received 20 mg/kg of Ruplizumab biweekly, followed by monthly doses, and demonstrated the efficacy of such treatment in significantly reducing anti-dsDNA antibody titers, decreasing hematuria, and increasing C3 complement fragment concentration. In another study, treating SLE patients with Ruplizumab eliminated their circulating CD38^+^ plasma cells and reduced their levels of anti-double stranded DNA, proteinuria, and Systemic Lupus Erythematosus Disease Activity Index (SLEDAI) [92]. Even a short-term treatment with this anti-CD154 mAb ameliorated serum complement concentrations and prevented hematuria in patients with LN [93]. Despite these promising results, the trial was terminated prematurely because of thromboembolic complications in some treated patients [93,94].

The other humanized anti-CD154 mAb, Toralizumab, is also composed of murine complementary-determining regions, although it binds to a different epitope than the ones used for Ruplizumab and is combined with human IgG1 heavy and light chains [94]. In a phase II clinical trial, patients with mild-to-moderate active SLE were randomized to receive six doses of Toralizumab (2.5 mg/kg to 10.0 mg/kg) for a period of 16 weeks. Results showed that SLEDAI scores had improved in all groups, without being significantly different than the placebo group. In addition, the type and frequency of adverse events observed in this trial were similar in both treatment and placebo groups [95]. These results and the occurrence of thromboembolic events in patients with Crohn’s disease, halted the progress of Toralizumab-based treatments in autoimmune or inflammatory diseases [3,105].

### 5.3. Second Generation Anti-CD154/CD40 Antibodies Overcoming Thromboembolic Complications

Thromboembolic complications observed in clinical trials of the above first-generation anti-CD154 mAbs are believed to result from platelet activation and aggregation following ligation of anti-CD154 mAb-sCD154 immune complexes to Fc gamma receptors located on platelets surface [3,106]. With the aim of reducing these side effects, second-generation Abs targeting CD154-mediated responses have recently been developed using Fc-independent mechanisms (Table 1).

Dapirolizumab pegol or CDP7657 (UCB Pharma) is a humanized anti-CD154 Fab fragment conjugated with polyethylene glycol (PEG), replacing its Fc region [3]. The use of a murine equivalent to CDP7657, consisting of a PEGylated monovalent Fab’ anti-murine CD154 Ab (MR1 Fab’ PEG), for the treatment of NZB/W F1 mice with active lupus induced disease remission [107]. In SLE patients, CDP7657 was shown to be well tolerated and was not associated with thromboembolic events [96]. Clinical trials also documented the improvement of disease activity following CDP7657 administration [97]. Further clinical trials in this context, more specifically a phase IIb study, showed that CDP7657 administration in patients with active SLE improved certain biological disease markers such as anti-dsDNA antibody titers [98]. The safety and efficacy of dapirolizumab pegol is currently being tested in two ongoing phase III clinical trials [108,109].

Another approach targeting the CD154/CD40 interaction is the use of antagonistic anti-CD40 mAbs. BI (Boehringer Ingelheim) 655064 is a humanized antagonistic non-depleting anti-CD40 mAb with a mutation at the Fc region abolishing its effector function [3,110]. Two phase I clinical trials assessing the efficacy, pharmacokinetics, and safety of BI 655 064 in healthy subjects revealed such agent to be well tolerated, not associated with thromboembolic complications, and interestingly, capable of inhibiting CD154 upregulation, thus having a high potential to abrogate CD154-CD40 interactions [99,100]. In addition, two phase II clinical trials investigating the use of BI 655064 in LN have recently been completed. Results seem to show a link between BI 655064 treatment and a reduction in SLEDAI total score in LN patients [3]. One of these studies suggested a potential beneficial effect (complete renal response as the endpoint outcome) of one of the doses used (180mg) in LN patients [101]. 

Iscalimab or CFZ533 (Novartis Pharma) is a fully human blocking non-depleting anti-CD40 mAb also mutated at its Fc region and incapable of stimulating Fc gamma receptors [111]. Studies on cynomolgus and rhesus monkeys with lupus demonstrated that treatment with CFZ533 induced a complete suppression of germinal center development in lymphoid organs, highlighting its capacity to inhibit CD154/CD40-induced pathways [112,113]. In a first-in-human phase I clinical trial, Iscalimab was shown to be well tolerated with no association with thromboembolic complications [102]. Considering these promising results, Iscalimab safety and efficacy were very recently tested in a phase II clinical trial in patients with active LN [103] and are also currently being tested in another phase II trial in SLE patients [104].

Given the overexpression of CD154 and CD40 on T and auto-reactive B cells [44], respectively in the renal interstitium, the contribution of the CD154/CD40 axis to immune complexes deposition at the level of renal tissues by enhancing activation of T and B cells and promoting the antigen-presentation function of DCs and monocytes, and the role of this axis in inducing pro-inflammatory functions of mesangial and renal tubular epithelial cells [56,60,61], it might be highly recommended to administer anti-CD154/CD40 agents to SLE patients with renal manifestations in an attempt to manage or even prevent the devastating clinical presentation of LN.

Targeting the CD154/CD40 pathway proved its therapeutic potential in yet other autoimmune diseases, providing further support for its use in SLE patients. Indeed, BI 655064 has been evaluated in patients with RA in a Phase IIa study. Results demonstrated decreased levels of inflammatory mediators, namely IL-6 and bone remodeling factors such as MMP-3 and RANK. Patients also exhibited a decrease in the percentage of activated CD95^+^ B cells and the concentration of IgG and IgA rheumatoid factor-positive auto-antibodies. In spite of promising biological and clinical changes in these patients, the study endpoint, which was defined as a 20% improvement of the RA score, was not met [114]. In the same line of evidence, the treatment of patients with Sjögren’s syndrome with Iscalimab reduced their disease activity index with a trend toward reduced auto-antibody response [115]. These studies, given their favorable safety profiles, support the development of further studies assessing the efficacy and safety of BI 655064 and Iscalimab in LN patients as described above.

## 6. Conclusions

The development of specific treatment options for SLE is critical to providing patients with better care and quality of life. Indeed, the standard practice for treatment or managing symptoms in SLE patients includes the use of intensive non-selective immunosuppressives. More recently, the use of targeted biologic therapies with better outcomes has surfaced; however, there remains an urgent need for more efficient treatment strategies, especially when devastating complications such as LN are manifested. The CD154-CD40 dyad, which plays an important role at different levels of SLE pathogenesis, has emerged as an interesting target for the development of novel biological therapies for disease treatment. Although first-generation Abs targeting this dyad were unsuccessful due to thromboembolic complications, second-generation Abs that lack the Fc region, an activator of FcRs on the platelet surface, are currently being tested in many clinical trials and may yield more promising results. However, these potential new treatment avenues do not take into account CD154 interactions with its recently discovered receptors belonging to the integrin family, which could also be highly involved in SLE pathogenesis and constitute important targets for therapeutic approaches. In this context, taking into consideration any pre-existing condition or SLE-related manifestation, which might be indicative of the CD154 interactions at play, is of importance. For instance, the anti-CD154 mAb, while interfering with all interactions of CD154, inhibits the binding of CD154 to the αIIbβ3 integrin on the surface of platelets. In the case of patients with atherosclerotic vessels, such inhibition promotes the instability and rupture of the atherosclerotic plaque leading to thrombotic events [4]. Thus, further investigation into the role of CD154-integrin dyads in SLE pathogenesis and the effect of blocking these interactions in SLE animal models and, ultimately, in patients with the disease should be urgently carried out. This may be an important research path for the better understanding of the implications of CD154 in SLE and for the identification of new specific targets for SLE treatment with better clinical outcomes.

## Figures and Tables

**Table 1:** Anti-CD154 and anti-CD40 agents in SLE clinical trials.

First Generation Anti-CD154 mAbs					
	Study Phase	Patients Condition	Outcome	Adverse Events	Refs.
Ruplizumab	Phase II	Patients with active LN	- reduced anti-dsDNA Abs - decreased hematuria - elevated C3 concentration - absence of CD38^+^ plasma cells in circulation - reduced proteinuria - improved SLEDAI	Thromboembolic complications	[92,93]
Toralizumab	Phase II	Patients with mild-to-moderate active SLE	- improved SLEDAI scores (but in study and placebo groups)	Thromboembolic complications	[3,94,95]
**Second generation anti-CD154 or anti-CD40 mAbs**					
Dapirolizumab pegol or CDP7657 (anti-CD154)	Phase I	SLE patients	- well tolerated - ameliorated SLEDAI	- not associated with thromboembolic complications	[96,97]
	Phase IIb	Patients with active SLE	- improved anti-dsDNA Ab titers		[98]
BI 655064 (antagonistic anti-CD40)	Phase I	Healthy subjects	- well tolerated - capable of inhibiting CD154 upregulation	- not associated with thromboembolic complications	[99,100]
	Phase II	Patients with LN	- reduction of SLEDAI - better renal response		[3,101]
Iscalimab or CFZ533 (antagonistic anti-CD40)	Phase I	Healthy subjects and RA patients	- well tolerated	- not associated with thromboembolic complications	[102]
	Phase II	SLE and LN patients	No publication yet (one study still ongoing)		[103,104]
